# Supplementary material for: The Systems Biology Research Tool: evolvable open-source software
Source: BMC Syst Biol. 2008 Jun 29;2:55. doi: 10.1186/1752-0509-2-55 (PMC2446383; doi:10.1186/1752-0509-2-55)
Supplement: Additional file 1 — SBRT Archive. An archive of the current version of the Systems Biology Research Tool. [file 1752-0509-2-55-S1.zip › sbrt-1.4.0/doc/developers_guide/api/sbrt/kernel/math/stat/comp/MannWhitneyU.html]

MannWhitneyU


|  |  |  |
| --- | --- | --- |
| **Overview** \| **Package** \| **Class** \| **Use** \| **Tree** \| **Deprecated** \| **Index** \| **Help** | |  |
| **PREV CLASS**   **NEXT CLASS** | **FRAMES**    **NO FRAMES**     **All Classes** |  |
| SUMMARY: NESTED \| FIELD \| CONSTR \| METHOD | DETAIL: FIELD \| CONSTR \| METHOD |  |


---


## sbrt.kernel.math.stat.comp Class MannWhitneyU

```
java.lang.Object
  sbrt.kernel.math.stat.comp.MannWhitneyU
```

---

``` public class MannWhitneyU extends java.lang.Object ```

This class is used to compute Mann-Whitney U statistics. The implementation is
based on the algorithm and equations provided on pages 428-430 in
Sokal, Robert and Rohlf, James (1995). Biometry, Third Edition. NY, W. H.
Freeman and Company; ISBN: 0-7167-2411-1.

**Author:**
:   This class was written and documented by Jeremiah Wright while
    in the Wagner lab.

---

**Constructor Summary**

`MannWhitneyU()`


| **Method Summary** | |
| --- | --- |
| `int` | `getN1(int size1, int size2)`             Returns the largest provided sample size. |
| `int` | `getN2(int size1, int size2)`             Returns the smallest provided sample size. |
| `double` | `getT(double uValue, int n1, int n2)`             Computes and returns an approximate t value for the provided U value. |
| `double` | `getU(double[] sampleA, double[] sampleB)`             Computes and returns the Mann-Whitney U statistic for the provided samples. |
| `double` | `getU(int[] sampleA, int[] sampleB)`             Computes and returns the Mann-Whitney U statistic for the provided samples. |
| `double` | `getU(java.util.List<? extends java.lang.Number> sampleA, java.util.List<? extends java.lang.Number> sampleB)`             Computes and returns the Mann-Whitney U statistic for the provided samples. |
| `static void` | `main(java.lang.String[] args)`             Used for testing. |

**Methods inherited from class java.lang.Object**

`clone, equals, finalize, getClass, hashCode, notify, notifyAll, toString, wait, wait, wait`

**Constructor Detail**

### MannWhitneyU

```
public MannWhitneyU()
```


**Method Detail**

### getU

```
public double getU(java.util.List<? extends java.lang.Number> sampleA,
                   java.util.List<? extends java.lang.Number> sampleB)
```

:   Computes and returns the Mann-Whitney U statistic for the provided samples.

    :   **Parameters:**: `sampleA` - a sample of numbers.: `sampleB` - a sample of numbers. **Returns:**: the Mann-Whitney U statistic for the provided samples.

---


### getU

```
public double getU(int[] sampleA,
                   int[] sampleB)
```

:   Computes and returns the Mann-Whitney U statistic for the provided samples.

    :   **Parameters:**: `sampleA` - a sample of integers.: `sampleB` - a sample of integers. **Returns:**: the Mann-Whitney U statistic for the provided samples.

---


### getU

```
public double getU(double[] sampleA,
                   double[] sampleB)
```

:   Computes and returns the Mann-Whitney U statistic for the provided samples.

    :   **Parameters:**: `sampleA` - a sample of doubles.: `sampleB` - a sample of doubles. **Returns:**: the Mann-Whitney U statistic for the provided samples.

---


### getT

```
public double getT(double uValue,
                   int n1,
                   int n2)
```

:   Computes and returns an approximate t value for the provided U value.
    This method uses the equation at the bottom of page 429 in Biometry.
    No correction for ties is made here.

    :   **Parameters:**: `uValue` - the computed U value.: `n1` - the size of the larger sample: `n2` - the size of the smaller sample **Returns:**: an approximate t value for the provided U value. **Throws:**: `java.lang.IllegalArgumentException` - if n1 < n2, or if n1 ≤ 20

---


### getN1

```
public int getN1(int size1,
                 int size2)
```

:   Returns the largest provided sample size.

    :   **Parameters:**: `size1` - a sample size.: `size2` - a sample size. **Returns:**: the largest provided sample size.

---


### getN2

```
public int getN2(int size1,
                 int size2)
```

:   Returns the smallest provided sample size.

    :   **Parameters:**: `size1` - a sample size.: `size2` - a sample size. **Returns:**: the smallest provided sample size.

---


### main

```
public static void main(java.lang.String[] args)
```

:   Used for testing.

    :   **Parameters:**: `args` - none required.


---


|  |  |  |
| --- | --- | --- |
| **Overview** \| **Package** \| **Class** \| **Use** \| **Tree** \| **Deprecated** \| **Index** \| **Help** | |  |
| **PREV CLASS**   **NEXT CLASS** | **FRAMES**    **NO FRAMES**     **All Classes** |  |
| SUMMARY: NESTED \| FIELD \| CONSTR \| METHOD | DETAIL: FIELD \| CONSTR \| METHOD |  |


---
